# Economic crisis, austerity and unmet healthcare needs: the case of Greece

**DOI:** 10.1186/s12913-016-1557-5

**Published:** 2016-07-27

**Authors:** Dimitris Zavras, Athanasios I. Zavras, Ilias-Ioannis Kyriopoulos, John Kyriopoulos

**Affiliations:** 1 Department of Health Economics, National School of Public Health, 196 Alexandras Avenue, 11521 Athens, Greece; 2 Department of Pediatric Dentistry, Goldman School of Dental Medicine, Boston University, 100 E Newton Street, Suite 706, Boston, MA 02118 USA; 3 Department of Social Policy, London School of Economics and Political Science, London, UK

**Keywords:** Austerity, Economic crisis, Healthcare, Unmet healthcare needs, Demand, Utilization, Greece

## Abstract

**Background:**

The programme for fiscal consolidation in Greece has led to income decrease and several changes in health policy. In this context, this study aims to assess how economic crisis affected unmet healthcare needs in Greece.

**Methods:**

Time series analysis was performed for the years 2004 through 2011 using the EU-SILC database. The dependent variable was the percentage of people who had medical needs but did not use healthcare services. Median income, unemployment and time period were used as independent variables. We also compared self-reported unmet healthcare needs drawn from a national survey conducted in pre-crisis 2006 with a similar survey from 2011 (after the onset of the crisis). A common questionnaire was used in both years to assess unmet healthcare needs, including year of survey, gender, age, health status, chronic disease, educational level, income, employment, health insurance status, and prefecture. The outcome of interest was unmet healthcare needs due to financial reasons. Ordinary least squares, as well as logistic regression analysis were conducted to analyze the results.

**Results:**

Unmet healthcare needs increased after the enactment of austerity measures, while the year of participation in the survey was significantly associated with unmet healthcare needs. Income, educational level, employment status, and having insurance, private or public, were also significant determinants of unmet healthcare needs due to financial reasons.

**Conclusions:**

The adverse economic environment has significantly affected unmet health needs. Therefore health policy actions and social policy measures are essential in order to mitigate the negative impact on access to healthcare services and health status.

## Background

In 2010, the Greek economy was placed under the surveillance of the European Commission, the European Central Bank, and the International Monetary Fund when the Hellenic Republic signed onto the first Economic Adjustment Programme (EAP) [1]. The programme included several fiscal measures and structural reforms aimed at reducing the general government and current account deficit and achieving public debt sustainability in the long run. The Greek economy entered a phase of severe recession, characterized by high unemployment and reduction of GDP [2]. The EAP included several measures, including significant wage and pension reductions as well as tax increases. Generally, the measures implemented since May 2010 can be characterized as a process of “internal devaluation” [3].

Several studies have noticed the adverse impact of economic crisis on health and healthcare. Specifically, recent publications suggest that the current economic crisis is associated with a drop in self-rated health status [4], a negative impact of the crisis on health trends [5] and difficulties on health promotion and public health policies [6]. Apart from the negative impact on health, economic crisis has adversely affected healthcare services [7, 8].

Unmet needs consist an indicator of equity and accessibility to healthcare services [9, 10] and they can be defined as *“the differences, if any, between those services judged necessary to deal appropriately with defined health problems and those services actually being received”* [11], while the scarcity of resources makes them inevitable [12].

During economic crises, the demand for healthcare services and the utilization of such services follows the general drop in socioeconomic status [13–15]. Such change may reflect barriers to access due to increased unemployment and reductions in disposable income [16, 17]. Even during periods of economic stability and growth, an individual’s inability to pay for healthcare services may result in unmet health needs [9]. Indeed, several studies indicate that unmet healthcare needs have increased in Greece [18], and it is expected that this trend will continue [19]. Generally, economic crises are associated with lower labour demand, disposable income reduction, problems on health financing and deterioration of access to healthcare [20]. Therefore, the research hypothesis of this study is associated with the extent to which unmet healthcare needs due to financial reasons have increased, and the characteristics of the socioeconomic groups that mainly face the unmet needs in Greece.

Estimating how unmet healthcare needs differ between periods of stability and periods of austerity is an under-studied question with real consequences for the health status of the population. In this context, the aim of the present study is to assess the impact of the economic crisis in Greece on the unmet healthcare needs.

## Methods

The analysis is based on annual time series data from the EU-SILC study from 2004 to 2011, which are publicly available [21]. The dependent variable was the percentage of people who had medical needs but did not use healthcare services. Independent variables were time [coded as 0:2004–2007 (no crisis), 1:2008–2009 (crisis starting, no austerity measures) and 2:2010–2011 (crisis with austerity measures in effect), median income, and unemployment. Initially we used the augmented Dickey-Fuller test to see if the variables were stationary. Given that the variables presented unit roots, we tested if the residuals of the ordinary least squares (OLS) model presented unit roots (without constant and trend). Since testing confirmed that regression residuals had no unit roots, regression was judged as non-spurious, and the variables co-integrated. A final model choice was based on the information criteria in AIC and BIC. We also tested for normality and heteroskedasticity of the residuals with the skewness and kurtosis test and the Breusch-Pagan/Cook-Weisberg test, as well as performed a link test to check for specification error. Finally, we tested for autocorrelation of standardized residuals via Durbin’s alternative test.

To study the effect of the financial crisis, controlling for other socioeconomic variables on the outcome of interest, namely the reason for unmet healthcare needs, we merged data from the two national surveys from 2006 to 2011 that were conducted by the National School of Public Health [22]. The sample sizes were 4003 in 2006 (n_2006_ = 4003), and 6569 in 2011 (n_2011_ = 6569, n_total 2006_ & _2011_ = 10,572), and they were both selected randomly based on stratification according to prefecture (based on the residence of the respondents), degree of urbanity based on NUTS II, age, and gender. Subjects were asked to report on experiences during the preceding year. Both surveys used a common questionnaire based on World Health Organization methodology [23] that had been validated in the past, and data collection involved a personal interview. In 2006 the interviews were conducted in the home of the respondents whereas in 2011 the interviews were conducted by telephone.

The final sample that we used for the analysis included 3120 patients who reported unmet needs. Specifically, 1243 of them were men (39.84 %), and the remaining 1877 were women (60.16 %), while the median age was 45 years. Among those who had stated that they have a medical need, 894 respondents (28.65 %) identified financial reason for not seeking care, whereas 2226 individuals (71.35 %) identified another reason for unmet healthcare needs. More details for the final sample used in the analysis are presented in Table 1.

**Table 1:** Distribution of unmet healthcare needs due to financial reasons per year

Year	Unmet healthcare needs due to financial reasons % (*n*)	Unmet healthcare needs due to other reasons % (*n*)	Total % (*n*)
2006	27.720 (349)	72.280 (910)	100 (1259)
2011	29.290 (545)	70.710 (1316)	100 (1861)
Total	28.650 (894)	71.350 (2226)	100 (3120)

The analysis focused on those participants who reported a medical or healthcare need, but no healthcare utilization. The outcome was dichotomized to 1 for unmet healthcare needs due to financial reasons, and to 0 for unmet healthcare needs due to other reasons. The final sample size was n_2006_ & _2011_ = 3120 (n_2006_ = 1259, n_2011_ = 1861). We gained permission to access this dataset from the Department of Health Economics, National School of Public Health.

Continuous variables were used as such, and Helmert coding was used for ordered variables, including education and income level. Various dummy variables were created for the nominal variables of employment and prefecture.

Statistical analysis was carried out in STATA 9.0. We used multiple logistic regression (MLR) to assess the effect of the main variable (year of participation) on the outcome (reason for unmet healthcare needs) controlling for various potential predictors or confounders. Potential predictors (independent variables) in the model were the following: a) gender (1: female, 2: male); b) age; c) self-reported health status (1: very bad, 2: bad, 3: medium, 4: good, 5: very good); d) existence of chronic health condition (1: no, 2: yes); e) education level (1: no education, 2: elementary school, 3: high school, 4: post high school and/or technical vocational education, 5: higher education, 6: university, 7: post-graduate education); f) income level (1: no income, 2: 1–500€, 3: 501–1000€, 4: 1001–1500€, 5: 1501–2000€, 6: 2001–3000€ and 7: 3001€+); g) employment status (1: working, 2: unemployed, 3: retiree, 4: homemaker 5: student or soldier, 6: other); h) public social security health insurance (1: yes, 2: no); i) private health insurance (1: yes, 2: no); j) urbanity status of permanent residence (1: rural, 2: urban); k) geographic prefecture (1: Attica, 2: East Macedonia and Thrace, 3: West Macedonia, 4: Central Macedonia, 5: Epirus, 6: Thessaly, 7: West Greece, 8: Central Greece, 9: Islands of Northern Aegean, 10: Islands of Southern Aegean, 11: Peloponnese, 12: Ionian Islands, 13: Crete); and l) year of survey (0: 2006, 1:2011). The appropriateness and fit of the final models were checked using several diagnostic methods, such as: i) link test, to test if the model suffers from specification error; ii) Hosmer and Leme show goodness of fit criterion; iii) skewness and kurtosis test of normality of the deviance residuals; and, iv) Brown and Forsythe test for the homoskedacity of the deviance residuals. ROC curves were fitted to explore the interpretation value of the models.

## Results

According to the model, the increase in unmet healthcare needs after the implemented austerity measures was statistically significant. In addition, according to the link test results, the model does not suffer from specification error (Table 2).

**Table 2:** Augmented Dickey-fuller test for unit roots

1 % Critical value	5 % Critical value	10 % Critical value
−3.750	−3.000	−2.630
Population Proportion with Unmet Healthcare Needs		
*p* = 0.876 (test statistic:-0.576)		
Year (2004–2007:0, 2008–2009:1, 2010–2011:0)		Year (2004–2007:0, 2008–2009:0, 2010–2011:1)
*p* = 0.462 (test statistic:-1.641)		*p* = 0.914 (test statistic:-0.378)
Median Income		
*p* = 0.461 (test statistic: −1.642)		
Unemployment (%)		
*p* = 0.997 (test statistic: 1.466)		

Based on the Augmented Dickey-Fuller test for unit roots, all the variables (year, median income, unemployment) we examined for the time series analysis presented unit roots (Table 3).

**Table 3:** OLS model results

Unmet healthcare needs	Coefficient	Std. Err.	t	*P* > t	95 % Confidence interval	
Year (2004–2007:0, 2008–2009:1, 2010–2011:0)	1.000	0.731	1.370	0.230	−0.880	2.880
Year (2004–2007:0, 2008–2009:0, 2010–2011:1)	2.150	0.731	2.940	0.032	0.270	4.030
Constant	6.400	0.422	15.160	0.000	5.315	7.485
AIC:2.780, BIC:5.840						

However, the final OLS model residuals (Table 4) did not present unit roots (Table 5). Thus, we conclude that the final model check led to co-integrated variables and to a regression model that is not spurious. Moreover, the model demonstrated a good fit, because the assumptions for the regression were satisfied. Residuals were found to follow a normal distribution (p_skewness_& _Kurtosis_ = 0.86) and residuals were homoskedastic (p_breusch-pagan/cook-weisberg_ = 0.85). In addition, Durbin’s alternative test (*p* = 0.77) indicates no serial correlation of the residuals.

**Table 4:** Augmented Dickey-fuller test for unit roots of the residuals

Test statistic	1 % Critical value	5 % Critical value	10 % Critical value
−3.145	−2.660	−1.950	−1.600

**Table 5:** Link test (OLS model)

Unmet healthcare needs	Coef.	Std. Err.	t	P > t	95 % Confidence interval	
hat	0.999	9.009	0.110	0.916	−22.159	24.159
hat^2^	1.37e-07	0.605	0.00	1.000	−1.556	1.556
constant	5.63e-06	33.050	0.000	1.000	−84.958	84.958

According to the MLR model, the year of participation was significantly associated with unmet healthcare needs due to financial reasons. More specifically, the odds of non-utilization of healthcare services due to financial reasons was 44 % higher in 2011 compared with 2006 (OR = 1.44), controlling for other socioeconomic predictors of utilization. Income, educational level, employment status and insurance were also significant.

Participants of the lower income group who were in need of medical care were 2.65 times more likely to be in front of unmet needs due to financial reasons, as compared with participants in the next income group (OR = 2.65). Similarly, the MLR model indicates that the lower the income, the higher the odds of unmet needs occurrence. The results are analytically described in Table 6.

**Table 6:** MLR model results

Unmet healthcare needs due to financial reasons	Odds ratio	Std. Err.	z	*P* > z	95 % Confidence interval	
Year of Study	1.441	0.147	3.580	0.000	1.179	1.761
Income (1 vs. 2+)	2.649	0.888	2.900	0.004	1.373	5.113
Income (2vs. 3+)	4.895	0.870	8.930	0.000	3.455	6.935
Income (3vs. 4+)	2.614	0.328	7.650	0.000	2.043	3.343
Income (4vs. 5+)	1.925	0.271	4.650	0.000	1.460	2.537
Income (5vs. 6+)	2.293	0.421	4.520	0.000	1.601	3.286
Income (6vs. 7)	2.152	0.621	2.660	0.008	1.223	3.788
Educational Level (1 vs. 2+)	1.728	0.415	2.280	0.023	1.080	2.765
Educational Level (2vs. 3+)	2.086	0.302	5.080	0.000	1.570	2.771
Educational Level (3vs. 4+)	1.268	0.158	1.900	0.057	0.993	1.620
Educational Level (4vs. 5+)	1.261	0.260	1.120	0.261	0.842	1.890
Educational Level (5vs. 6+)	1.034	0.234	0.150	0.883	0.664	1.611
Educational Level (6vs. 7)	0.708	0.225	−1.090	0.278	0.379	1.321
Unemployed	1.490	0.252	2.360	0.018	1.070	2.076
Retired	1.094	0.141	0.690	0.488	0.849	1.408
Homemaker	1.301	0.180	1.920	0.055	0.994	1.703
Student, Soldier	0.726	0.186	−1.250	0.211	0.440	1.199
Other Occupation	0.137	0.146	−1.870	0.062	0.017	1.104
Public Insurance	0.448	0.096	−3.730	0.000	0.294	0.683
Private Insurance	0.715	0.111	−2.150	0.031	0.526	0.970
constant	0.723	0.168	−1.400	0.162	0.459	1.139

Moreover, higher likelihood of not expressing the need into utilization due to financial reasons was noted for subjects that were illiterate (OR = 1.73) or for subjects that had received elementary school education (OR = 2.09), as compared with participants who reported higher education. It is also noteworthy that the employment status affects the likelihood of unmet needs due to financial reasons. Specifically, the odds of unmet needs due to financial reasons for unemployed increased by 49 % compared with people who were employed and in need of care at the time of the interview.

Additionally, the presence of insurance (public or private) is also statistically significant, that is, insurance offers protection against lack of healthcare utilization. Having an unmet healthcare need due to financial reasons was lower among those individuals with private insurance (OR = 0.71) and even lower among those with public insurance (OR = 0.45). Details are presented in Table 6.

**Fig. 1:**
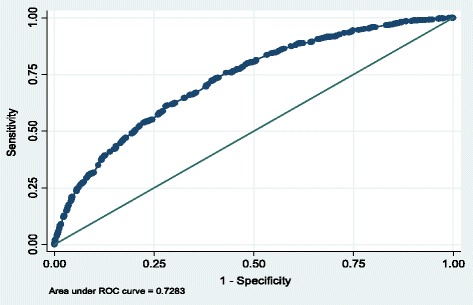
ROC curve

Model diagnostics via the link test revealed that the model does not suffer from specification error since hat is statistically significant but hat^2^ is not statistically significant, as shown in Table 7. The ROC curve revealed that the model’s interpretation value is acceptable since the area under the curve is 0.73 (Fig. 1). Further, the model demonstrated a good fit since the *p*-value for Hosmer-Lemeshow was 0.92. Assumptions for the regression were satisfied; deviance residuals were found to follow a normal distribution (p_skewness_& _kurtosis=_0.09 > 0.05), with constant variance (p_brown_ & _foresythe_ = 0.73 > 0.05).

**Table 7:** Link test (MLR model)

Unmet healthcare needs due to financial reasons	Coef.	Std. Err.	z	*P* > z	95 % Confidence interval	
hat	0.963	0.091	10.630	0.000	0.785	1.140
hat^2^	−0.027	0.051	−0.530	0.599	−0.126	0.073
constant	0.004	0.061	0.070	0.947	−0.115	0.123

## Discussion

The aforementioned suggest that unmet healthcare needs increased after the enactment of austerity measures in Greece, mainly due to patients' difficulty to cover the costs of medical care. Generally, the cost of medical care is regarded as one of the main predictors of non-utilization [24, 25].

As mentioned previously, the odds of unmet needs due to financial reasons were 44 % higher in 2011 as compared with 2006. It is noteworthy that this finding is consistent with previous findings, which mention that healthcare utilization drops during economic crises, especially because of the presence of financial barriers [26, 27]. Recent findings from Greece also mention that financial barriers in access for chronic patients increased during the period of the economic crisis [28].

Generally, our results imply that unmet medical needs due to financial reasons are associated with income and health insurance, namely that low-income individuals, unemployed and uninsured are more likely to face unmet needs.

Low socioeconomic status is significantly associated with unmet needs due to financial reasons. Indicatively, we found that the lower the income, the higher the odds of unmet medical needs due to financial reasons. Another study has also found that income is considered as an important determinant of health services utilization in Greece, by estimating the income elasticity of utilization [29].

The impact of educational level is present but limited, given that it presents statistical significance on lower educational level, but not to the higher ones. A recent publication has also reported that individuals who have accomplished post-secondary education are associated with lower odds of unmet needs due to financial reasons in Greece [30].

Similarly, the presence of health insurance leads to lower odds of unmet healthcare needs. These findings are in line with several publications, which indicate that the absence of health insurance presents a strong correlation with unmet health needs [12, 31, 32].

In accordance with the aforementioned, many studies have noted the relationship between healthcare services utilization and income, education, employment status, or characteristics of health system (such as health insurance) [33–36]. Moreover, low socioeconomic status has also been found to predict non-utilization of healthcare services [37, 38].

In addition, the present analysis examined the effect of gender, age, and health-related variables (self-reported health status, existence of a chronic disease) on healthcare utilization. While the above variables determine the degree of need [39], none of them presented a statistically significant relationship with unmet healthcare needs due to financial reasons. In a similar fashion, degree of urbanity and prefecture, variables that may be used as proxies for access to care, did not have a significant effect. However, Kentikelenis et al. have found that gender and urbanity affects the odds of facing unmet needs due to financial reasons [30].

This analysis implies that economic aspects constitute the forefront of healthcare utilization. Specifically, income, unemployment and uninsurance are key variables affecting the probability of unmet health needs occurrence. If the economic variables were not the predominant predictors of unmet needs, one would expect non-economic variables such as the existence of a chronic disease to lead to significantly higher odds of utilization among low socioeconomic subjects. This specific finding is strengthened by empirical observations documenting the relationship between self-reported health status, socioeconomic status, and the negative impact that financial crises exert at the population’s health status [40, 41].

Our findings validate previous findings reported in the existing literature, which reports that a need for care in segments of the population is not expressed during periods of economic crisis and recession [42]. The phenomenon of non-utilization of healthcare services among those in need of care seems to reflect reduction in disposable income due to unemployment and drastic cuts in salaries and pensions [43, 44]. This inability to seek care ultimately leads to poverty, social marginalization and adverse effects on health [45].

Unmet healthcare needs and access to healthcare constitute a significant issue that should be addressed in Greece. However, a significant question relates to how unmet healthcare needs potentially affect health outcomes. Generally, it is widely acknowledged that healthcare services constitute a limited predictor of health outcomes. Apart from access and use of health services, the determinants of health include income, education, social status, lifestyle, physical environment, social support networks, genetics, and gender [46]. There is ample evidence about the effects of economic downturn on health, in Greece [4, 5, 19] and internationally [44, 47]. Therefore, although our findings illustrate a problematic dimension regarding healthcare service, the extent of the adverse impact of unmet healthcare needs on health outcomes remains unanswered.

As with any study of this kind, the present analysis has limitations. For instance, the analysis does not capture the period 2012–2015, during which there was deep recession and several measures were implemented. Moreover the time series consists of limited observations. Ideally, panel data analysis would be technically preferable, however there is not a comprehensive database for such an analysis. It is also noteworthy that self-reported unmet health needs are an indicator of access to healthcare; however the extent to which unmet needs are associated with barriers to access or individual preferences is a crucial aspect. Therefore, several other access indicators should also be examined for obtaining an holistic view regarding access to healthcare [9]. Another conceptual limitation in the studies of this kind relates to what is meant by “healthcare needs”. In this case, the data were derived by Eurostat and the National School of Public Health Survey. Both examined the self-reported unmet healthcare needs.

## Conclusions

According to the aforementioned, unmet health needs due to financial reasons have increased during the last years. Moreover, the odds of facing unmet health needs are higher for unemployed, uninsured and low-income patients. Therefore, given that this period is characterized by high unemployment, uninsurance and low incomes, targeted social policy measures towards the vulnerable population groups are timely and of great importance. In addition, the role of health policy is crucial in terms of reducing the adverse impact of unmet health needs and the potential implications on access to medical care and population health.

## Abbreviations

AIC, Akaike Information Criterion; BIC, Bayesian Information Criterion; EAP, Economic Adjustment Programme; EU-SILC, European Union Statistics on Income and Living Conditions; GDP, Gross Domestic Product; OLS, ordinary least squares; OR, odds ratio
